# Evolutionary insights on critically endangered Kashmir red deer or hangul (*Cervus hanglu hanglu*) through a mitogenomic lens

**DOI:** 10.7717/peerj.15746

**Published:** 2023-10-19

**Authors:** Khursheed Ahmad, Ankit Shankar Pacha, Rashid Yahya Naqash, Sathish Kumar Peddamma, Srinivas Yellapu, Shenu Hudson, Dushyant Singh Baghel, Parag Nigam, Samrat Mondol

**Affiliations:** 1 Sher-e-Kashmir University of Agricultural Sciences and Technology of Kashmir, Srinagar, Jammu and Kashmir, India; 2 Animal Ecology and Conservation Biology, Wildlife Institute of India, Dehradun, Uttarakhand, India; 3 Department of Wildlife Protection, Jammu & Kashmir Government, Srinagar, Jammu and Kashmir, India; 4 Nucleome Informatics Pvt. Ltd., Hyderabad, Telangana, India

**Keywords:** Tarim red deer, Endemism, Next-generation sequencing, Phylogeny, Divergence time, Large herbivore conservation

## Abstract

**Background:**

The Kashmir red deer or Hangul (*Cervus hanglu hanglu*) is the only Tarim red deer species endemic to India. With a current estimated population size of fewer than 200 individuals, this critically endangered species is confined to the greater Dachigam landscape in Jammu and Kashmir. Poaching, habitat loss and fragmentation, resource competition with livestock, and small population size are the major conservation challenges for this species.

**Methods:**

Blood sampling was conducted from two wild Hangul individuals during radio-collaring operations at Dachigam National Park, Kashmir in 2013 and 2020, respectively. Using next-generation sequencing approach, we sequenced the 16,351 bp long mitogenome of two wild-caught Hangul individuals (1 M:1 F at ~14× and ~10× coverage, respectively) from Dachigam National Park.

**Results:**

The annotated sequences were identical with an AT-rich composition, including 13 protein-coding genes (11,354 bp), 22 tRNA genes (1,515 bp), two ribosomal genes (2,526 bp) and a non-coding control region (917 bp) in a conserved order like other red deer species. Bayesian phylogenetic reconstruction of the red deer complex revealed two major groups: the elaphoid and the wapitoid clades. Hangul formed a distinct clade with its other subspecies *C. hanglu yarkandensis* and is sister to the Hungarian red deer (*C. elaphus hippelaphus*). Divergence time analyses suggested that the Tarim deer species group separated ~1.55 Mya from their common ancestors and Hangul diverged ~0.75 Mya from closely related *C. yarkandensis*, corroborating with the known paleobiogeographic events related to refugia during glaciations in the Pleistocene era. This study provides baseline information on Hangul mitogenome for further research on phylogeography and other population parameters and helps in developing suitable conservation plans for this species.

## Introduction

The Kashmir red deer or Hangul (*Cervus hanglu hanglu*) is the only member of the Tarim red deer complex that is endemic (Brook et al., 2017) to the Indian subcontinent. Historically distributed across high altitudes of Kashmir, valleys of Chenab River and upper Himachal Pradesh, this subspecies is currently confined to the greater Dachigam landscape in the north-east of Srinagar, Jammu and Kashmir (Ahmad, Sathyakumar & Qureshi, 2009; Ahmad & Nigam, 2014) with only viable population found in Dachigam National Park (Ahmad, Sathyakumar & Qureshi, 2009; Ahmad & Nigam, 2014; Mukesh et al., 2015). With a current global population size of <200 individuals (reduced from ~3,000–5,000 animals during 1990s) and decreasing trend, Hangul is currently considered as ‘Critically endangered’ by IUCN (under C1 criteria) (Brook et al., 2017) and ‘Schedule 1’ under the Indian Wildlife (Protection) Act, 1972 (*i.e*., the highest protection status). The major concerns for this subspecies across its distribution are small population size, low fawn-female ratio, reduced calf survival (biological factors) and poaching, habitat loss and fragmentation, and competition for resources from livestock (anthropogenic factors) (Ahmad, Sathyakumar & Qureshi, 2009; Ahmad & Nigam, 2014; Mukesh et al., 2015).

Phylogenetically Hangul belongs to the red deer complex which has been extensively studied because of their global distribution (Ludt et al., 2004; Lorenzini & Garofalo, 2015; Kumar et al., 2017; Meiri et al., 2018; Mackiewicz et al., 2022). Recent genetic analyses suggest existence of two red deer groups: the eastern/Wapiti (consisting of species distributed across eastern Asia and North America) and western/Tarim deer (species ranging across Europe, North Africa, Middle East, and central Asia) (Lorenzini & Garofalo, 2015; Kumar et al., 2017; Meiri et al., 2018; Mackiewicz et al., 2022). Hangul, despite being genetically identified as a separate group within this species complex till date has not been studied in detail. While information on population status, distribution and conservation challenges is available (Ahmad, Sathyakumar & Qureshi, 2009; Ahmad & Nigam, 2014; Mukesh et al., 2015), genetic analyses related to their phylogenetic position and divergence is confined to single marker-based studies, single species within group (*Cervus hanglu yarkandensisr*) or inconclusive (Ludt et al., 2004; Lorenzini & Garofalo, 2015; Kumar et al., 2017; Meiri et al., 2018; Hu et al., 2019; Doan et al., 2022; Mackiewicz et al., 2022), possibly due to lack of sufficient data. In this study, we present next generation sequencing based whole mitogenome data and combine the information with already available genetic data to assess phylogenetic position of Hangul among central Asian red deer clade and its sister groups. In addition, we also provide insights on its divergence period and compare our results with available information. We believe that such detailed analyses with mitogenome would help us in generating valuable information on its evolution, genetic health and therefore assist in developing appropriate conservation plans for the isolated and small populations of this species.

## Methods

### Research permits and ethical considerations

All required research permissions towards fieldwork and biological sampling were accorded by the Chief Wildlife Warden, Department of Wildlife Protection, Jammu and Kashmir (Permit Nos. WLP/TECH/116-19, WLP/TECH/871-74/2013, and WLP/F-101/Res/2019-20/424-27). Blood samples (*n* = 2) were collected as part of an ongoing study titled “Long-term conservation plan for Hangul: movement pattern study using satellite telemetry”, where invasive sampling was conducted during radio-collaring operations at Dachigam National Park, Kashmir in 2013 and 2020, respectively. Ethical permissions were provided by the Sher-e-Kashmir University of Agricultural Sciences and Technology of Kashmir, Srinagar, Jammu and Kashmir, India (letter no. AU/FVS/PS-23/22/8553).

### Sampling

Apparently healthy adult Hangul (*n* = 2, 1 M:1 F) individuals captured for satellite collaring were sampled in this study. The male was captured in 2013 (estimated weight of ~150 kg) whereas the female was captured in 2020 (estimated weight of ~120 kg). In both cases, the animals were lured into an open forest patch using green fodder and salt and were chemically immobilized using a combination of medetomidine (10 mg/ml, Wildlife Pharmaceuticals Inc., Fort Collins, CO, USA) and ketamine hydrochloride (100 mg/ml, Troy laboratories Pvt. Ltd., Glendenning, NSW, Australia) at dose rates of 90 μg kg^−1^ and 1.45 mg kg^−1^ for male and 120 μg kg^−1^ and 2.08 mg kg^−1^ for female, respectively (Jalanka, 1988; Jalanka & Roeken, 1990). Drug delivery was carried out remotely from a hide using Dan-inject syringe projector (Model-IM) from a distance of 30–40 m. The animals were approached after achieving sedation safe for handling, and blood sampling was conducted. Blood was collected aseptically (5 ml from each animal) by jugular vein puncture in EDTA BD Vacutainer (BD Diagnostics, Franklin Lakes, NJ, USA) and transported to the laboratory within 2 h and stored at −20 °C till further processing. Drug reversal was assisted by administering 5 mg/ml atipamezole (Dechra, Northwich, United Kingdom) intramuscularly at a dose rate of 0.2 mg kg^−1^ body weight following completion of necessary procedures.

### DNA extraction and mitogenome sequencing

Mitogenome sequencing of Hangul was performed using next-generation sequencing (NGS) approach. Genomic DNA was extracted from two blood samples using MN NucleoSpin Blood kit (Macherey-Nagel, Duren, Germany) following manufacturers’ instructions. DNA quality was checked through 1% agarose gel electrophoresis and Qubit 3.0 fluorometer (Invitrogen, Life Technologies, Waltham, MA, USA). Following this, paired-end libraries were prepared using NEBNext Ultra DNA Library Prep kit (New England Biolabs, Ipswich, MA, USA) where the genomic DNA was enzymatically fragmented, end-polished, A-tailed, and ligated with full length Illumina sequencing adapters. The PCR amplification was conducted using universal P5 and indexed P7 oligos with following conditions: initial denaturation at 98 °C for 30 s, followed by four cycles of denaturation (98 °C for 10 s), annealing (65 °C for 75 s) and final extension (65 °C for 5 min). The constructed libraries were checked for purity, size, and concentration using Agilent 2100 Bioanalyzer (Agilent Technologies, Santa Clara, CA, USA). Finally, the qualified libraries (2 × 150 bp) were sequenced on Illumina NovaSeq6000 (Illumina, San Diego, CA, USA) platform at the Next Generation Genomics Facility of Nucleome Informatics Pvt. Ltd, Hyderabad, India. The raw sequence reads were screened using FastQC (https://www.bioinformatics.babraham.ac.uk/projects/fastqc/) for quality assurances and further analysed using FastP ver 0.20.1 (Chen et al., 2018) to remove the adapter sequences, low-quality reads (Q < 20) and reads with <30 bp length. After quality filtering, high-quality reads were assembled using animal mtDNA database sequences integrated in GetOrganelle ver 1.7.5 (Jin et al., 2020). The process involved following steps: (a) mapping reads to seed sequence against database using Bowtie 2 (Langmead & Salzberg, 2012), (b) conducting *de novo* assembly using SPAdes with different k-mer values (Bankevich et al., 2012), and (c) accurate identification of target organelle contigs. The assembled mitogenomes were validated by mapping against the red deer (*Cervus elaphus*) mitogenome (CerEla 1.0, OU343111.1).

### Data analysis

#### Hangul mitogenome annotation

The sequenced Hangul mitogenomes (*n* = 2) were annotated using MITOS2 web (Bernt et al., 2013) for structural information (coding regions, incomplete stop codons, overlaps *etc*.). Mitogenome map was generated using OGDRAW (Greiner, Lehwark & Bock, 2019) while skew analysis was performed using the following formula: GC skew = (G−C)/(G+C); AT skew = (A−T)/(A+T) to assess any possible nucleotide composition bias. Two sequences were aligned using MEGA v7 (Kumar, Stecher & Tamura, 2016) to check any differences between the male and female mitogenome data. Further, nucleotide percentages and codon usages were calculated in MEGA v7 (Kumar, Stecher & Tamura, 2016).

#### Comparative mitogenome analyses and hangul phylogeny

To ascertain Hangul’s phylogenetic position and estimate its divergence time, all available mitogenomes of Hangul (MW430050 and MW430051), other cervids (*n* = 13 species) and one sequence each of Muntiacini and Odocoileini were downloaded (Wada, Nishibori & Yokohama, 2007; Pan et al., 2014; Frank et al., 2016) from GenBank (see Table 1). Upon scrutiny, it was observed that both Hangul mitogenomes from GenBank were identical and therefore only one of the sequences (MW430051) was used in all analyses. Three sequences from Bovidae group: wild buffalo (*Bubalus arnee*) (Pacha et al., 2021), Indian gaur (*Bos gaurus*) (Hassanin et al., 2012) and Cape buffalo (*Boselaphus tragocamelus*) (Hassanin et al., 2012) were used as outgroup in this analysis. MEGA v7 was used to estimate inter-sequence mean pairwise genetic distance (Kumar, Stecher & Tamura, 2016).

**Table 1:** Nucleotide composition comparison with other red deer species.

Species	Common names	Accession no.	Complete mitogenome			Protein coding region (PCG)			rRNA			tRNA				Control region		
			Length (bp)	AT skew	GC skew	Length (bp)	AT skew	GC skew	Length (bp)	AT skew	GC skew		Length (bp)	AT skew	GC skew	Length (bp)	AT skew	GC skew
*Cervus hanglu hanglu*	Kashmir stag		16,351	0.072	−0.289	11,354	0.042	−0.360	2,526	0.218	−0.097		1,515	0.027	−0.135	917	−0.038	−0.213
*Cervus hanglu yarkandensis*	Yarkand deer	GU457435	16,351	0.072	−0.291	11,354	0.042	−0.358	2,525	0.216	−0.089		1,515	0.116	−0.144	918	−0.044	−0.199
*Cervus elaphus hippelaphus*	Hungarian red deer	KT290948	16,354	0.072	−0.291	11,354	0.043	−0.361	2,526	0.212	−0.087		1,515	0.113	−0.132	920	−0.065	−0.202
*Cervus canadensis songaricus*	Tian Shan wapiti	KJ025072	16,419	0.072	−0.288	11,354	0.044	−0.360	2,514	0.214	−0.091		1,516	0.119	−0.139	996	−0.019	−0.184
*Cervus canadensis kansuensis*	Gansu wapiti	NC039923	16,430	0.072	−0.286	11,354	0.042	−0.358	2,526	0.216	−0.091		1,516	0.117	−0.136	995	−0.030	−0.160
*Cervus canadensis*	Elk	MT534583	16,428	0.072	−0.288	11,354	0.042	−0.358	2,526	0.216	−0.089		1,516	0.119	−0.144	993	−0.024	−0.172
*Cervus canadensis nannodes*	Tule elk	MT430939	16,428	0.072	−0.288	11,354	0.042	−0.358	2,526	0.216	−0.089		1,516	0.119	−0.144	993	−0.024	−0.172
*Cervus nippon hortulum*	Dybowski’s sika deer	KR868807	16,421	0.074	−0.289	11,354	0.045	−0.361	2,529	0.217	−0.084		1,515	0.122	−0.147	984	−0.042	−0.160
*Cervus nippon sichuanicus*	Sichuan sika deer	JN389443	16,429	0.076	−0.291	11,354	0.045	−0.363	2,528	0.226	−0.106		1,517	0.118	−0.140	991	−0.048	−0.160
*Cervus nippon kopshi*	South china sika deer	HQ832482	16,429	0.074	−0.291	11,354	0.047	−0.365	2,527	0.223	−0.104		1,517	0.118	−0.140	992	−0.048	−0.166
*Cervus nippon yakushimae*	Yakushima sika deer	AB218689	16,440	0.077	−0.295	11,354	0.047	−0.368	2,532	0.221	−0.102		1,515	−0.038	−0.071	998	−0.038	−0.177
*Cervus nippon yeonsis*	Yezo sika deer	AB210267	16,543	0.079	−0.295	11,354	0.047	−0.365	2,526	0.222	−0.097		1,515	0.128	−0.164	1,109	−0.005	−0.170
*Cervus nippon centralis*	Sika deer	NC006993	16,663	0.077	−0.295	11,354	0.045	−0.365	2,524	0.222	−0.099		1,515	0.123	−0.161	1,231	−0.012	−0.152

A two-parameter substitution model (GTR+G, decided using jModelTest (Darriba et al., 2012)) along with a gamma distribution of evolutionary rates (across sites), and default shape parameter setting were used in the phylogenetic done *via* MrBayes v 3.2.7 (Ronquist et al., 2012). The MCMC parameters incorporated two runs of four chains (1 million generations each with sampling after 1,000 generations) till split frequencies were less than 0.01. For each node posterior probabilities were calculated. To estimate clade-specific divergence of *Cervus hanglu*, birth-death speciation was considered as tree prior with uncorrelated relaxed log normal clock (Ritchie, Lo & Ho, 2017; Steiner, Houck & Ryder, 2018) in BEAST v.2.3.0 (Drummond & Rambaut, 2007). During analysis, four fossil based internal node calibration points were employed with normal distribution priors: (i) root age for the split of Cervidae and Bovidae at 18 ± 2 Mya, (ii) Cervini-Muntiacini split around 9 ± 1 Mya, and (iii) oldest fossil of tribe Odocoileini to be 5 ± 1 Mya (Zhang & Zhang, 2012). The Cervini group was considered under monophyletic constraints. MCMC runs for tMRCA (the Most Recent Common Ancestor) inference involved 100 million generations, sampled at every 10,000 states with 10% burn-in. Data convergence was analysed using Tracer v. 1.5 (Rambaut & Drummond, 2007) and the divergence time tree which showed maximum clade credibility was inferred with TreeAnnotator (Helfrich et al., 2018) and visualised in FigTree v.1.4.2 (Rambaut, 2009). To assess the effects of data size in determining the patterns of phylogenetic relationship and divergence time the entire analyses were conducted using two additional data set: (a) with complete Cyt b and (b) complete Control region as these two markers have earlier been used in Hangul phylogeny work.

## Results

### Mitogenome organisation

A total of 215.84 million (female sample) and 284.22 million (male sample) sequence reads were generated through NGS approach. For mitogenome assembly, following paired and unpaired mapped reads were used: female-50,466 and 3,070; male-114,948 and 5,152. After data filtering, 213.7 million (female, 99.02%) and 281.78 million (male, 99.1%) of quality reads were obtained. The assembly lead to ~10× (female, 29.8 GB data) and ~13.74× (male, 39.3 GB data) coverage of Hangul mitogenome. The assembled mitogenome of Hangul generated in this study was of 16,351 bp length and matched with the available Hangul and other species reference sequences. The GenBank accession numbers for the sequences are ON416884 (female) and ON416885 (male), respectively.

The annotated Hangul mitogenome showed conserved gene order like other red deer species *i.e*. protein-coding genes (PCGs, *n* = 13), tRNA genes (*n* = 22), ribosomal genes (*n* = 2) and a non-coding control region (Table 2). Out of these, 29 genes are encoded on the heavy strand (H-strand) and remaining nine (NADH6 and eight tRNA genes: tRNA^Gln^, tRNA^Asn^, tRNA^Ala^, tRNA^Cys^, tRNA^Tyr^, tRNA^Ser^, tRNA^Glu^ and tRNA^Pro^) are encoded on the light strand (L-strand). The number of overlapping regions (*n* = 13) and intergenic spaces (*n* = 15) were also consistent with other red deers (Table 2). NADH1, NADH2, NADH4L, NADH5, two cytochrome oxidases (COII and COIII), ATP8 and ATP6 and O_L,_ showed overlaps ranging from 1 to 40 bp. The tRNA^Leu^ gene showed the largest overlap (9 bp) with NADH5 whereas three tRNA genes (tRNA^Ile^, tRNA^Thr^, tRNA^Gly^) showed the smallest overlap (1–3 bp) (Table 2). Similarly, the intergenic spaces ranged between 1–7 bp, with the longest between tRNA^Ser^ and tRNA^Asp^, respectively. The O_L_ was 31 bp long and found in the WANCY region between tRNA^Asn^ and tRNA^Cys^. Overall, the nucleotide composition comprised of 28.8% T, 24.5% C, 33.3% A and 13.5% G, with a positive AT value (0.072) (and a negative GC value of −0.289), indicating a AT-rich Hangul mitogenome (Table 1).

**Table 2:** Organisation of mitochondrial genome in *Cervus hanglu hanglu*.

Genes	Position				Codons		Strand
	Start	Stop	Length	Spaces\overlap	Start	Stop	
tRNA^Phe^ (GAA)	1	69	69	0			H
rrnS	70	1,025	956	0			H
tRNA^Val^ (TAC)	1,026	1,092	67	0			H
rrnL	1,093	2,662	1,570	1			H
tRNA^Leu^ (TAA)	2,664	2,738	75	2			H
nad1	2,741	3,697	957	−1	ATG	TAA	H
tRNA^Ile^ (GAT)	3,697	3,765	69	−3			H
tRNA^Gln^ (TTG)	3,763	3,834	72	2			L
tRNA^Met^ (CAT)	3,837	3,905	69	0			H
nad2	3,906	4,949	1,044	−2	ATA	TAG	H
tRNA^Trp^ (TCA)	4,948	5,015	68	2			H
tRNA^Ala^ (TGC)	5,018	5,086	69	1			L
tRNA^Asn^ (GTT)	5,088	5,160	73	2			L
OL	5,163	5,193	31	−1			H
tRNA^Cys^ (GCA)	5,193	5,260	68	0			L
tRNA^Tyr^ (GTA)	5,261	5,329	69	1			L
cox1	5,331	6,875	1,545	−3	ATG	TAA	H
tRNA^Ser^ (TGA)	6,873	6,941	69	7			L
tRNA^Asp^ (GTC)	6,949	7,016	68	1			H
cox2	7,018	7,701	684	3	ATG	TAA	H
tRNA^Lys^ (TTT)	7,705	7,772	68	1			H
atp8	7,774	7,974	201	−40	ATG	TAA	H
atp6	7,935	8,615	681	−1	ATG	TAA	H
cox3	8,615	9,399	785	−1	ATG	TA(A)	H
tRNA^Gly^ (TCC)	9,399	9,467	69	0			H
nad3	9,468	9,814	347	−10	ATA	TA(A)	H
tRNA^Arg^ (TCG)	9,815	9,883	69	0			H
nad4l	9,884	10,180	297	−7	ATG	TAA	H
nad4	10,174	11,551	1,378	0	ATG	T(AA)	H
tRNA^His^ (GTG)	11,552	11,620	69	0			H
tRNA^Ser^ (GCT)	11,621	11,680	60	1			H
tRNA^Leu^	11,682	11,751	70	−9			H
nad5	11,743	13,572	1,830	−17	ATA	TAA	H
nad6	13,556	14,083	528	0	ATG	TAA	L
tRNA^Glu^ (TTC)	14,084	14,152	69	4			L
cytb	14,157	15,296	1,140	3	ATG	AGA	H
tRNA^Thr^ (TGT)	15,300	15,369	70	−1			H
tRNA^Pro^ (TGG)	15,369	15,434	66	167			L
OH	15,602	16,145	544	206			H
D-Loop	15,435	16,351	916	/			H

### Protein coding genes

Hangul mitochondrial protein-coding genes (PCGs) was 11,354 bp in length with an AT-skewed base composition of 29.7% T, 25.9% C, 32.3% A and 12.2% G (Table 1). Out of the 13 coding genes majority (*n* = 12) were on the H-stand. These included six NADH genes (NADH1, NADH2, NADH3, NADH4, NADH5, NADH6 and NADH4L), ATPases (ATP6 and ATP8), cytochrome c oxidases (COI, COII and COIII) and cytochrome b (Cyt b). NADH4L was coded on the L-strand consistent with mitogenome organisation of other red deer species. NADH2, NADH3 and NADH5 have ATA start codon compared to others that have ATG. Only Cyt b (AGA), NADH2 (TAG), NADH4 and COIII had incomplete stop codon (T_ _) at the 5′ terminal of the adjacent gene to be completed during post transcription modification *i.e.* polyadenylation process. The Relative Synonymous Codon Usage (RSCU) for all PCGs consist of 3,784 codons.

### rRNA, tRNA genes and control region

**Figure 1:**
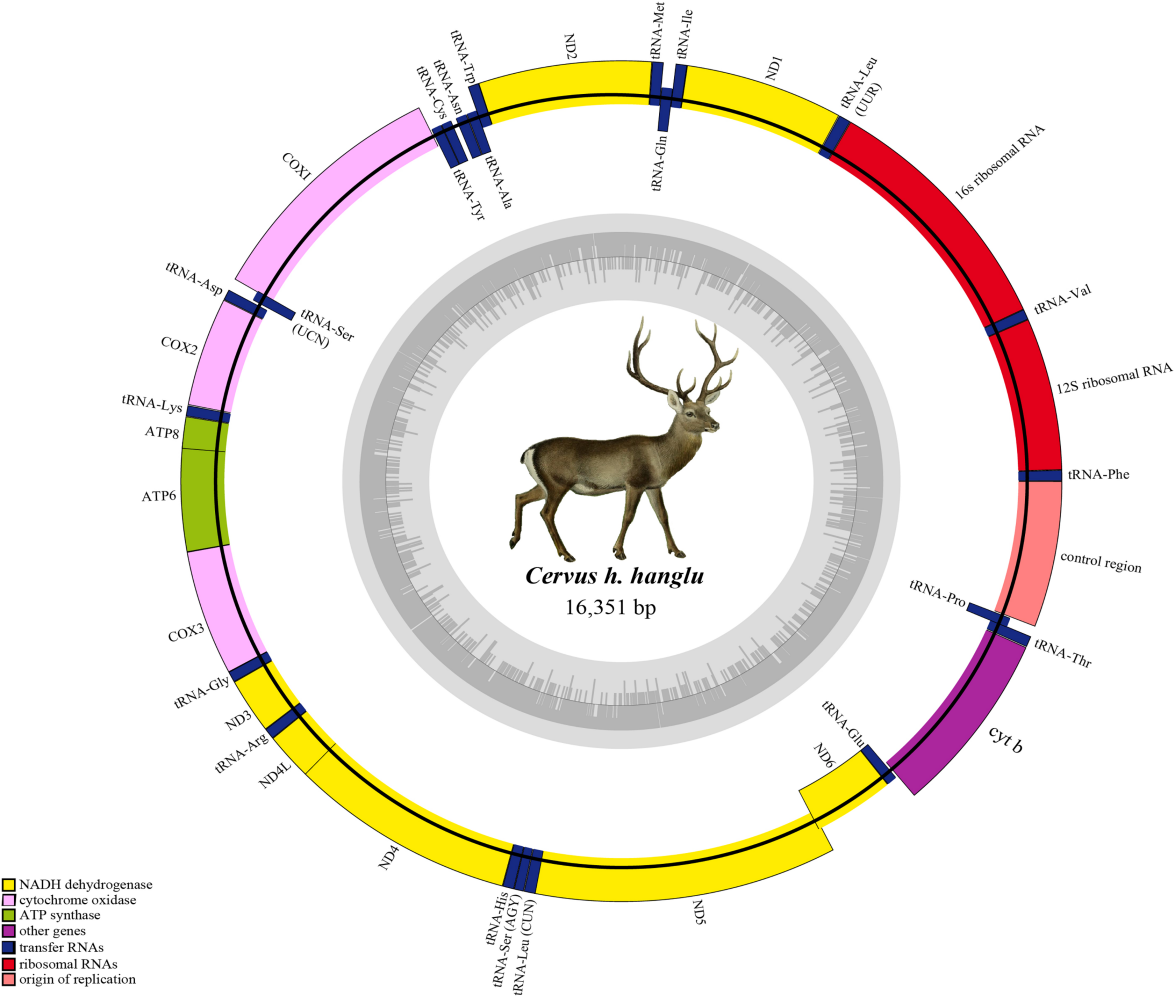
Complete mitochondrial genome of Hangul (*Cervus hanglu hanglu*) with location of genes. The genes in the heavy strands are shown outside. The inner grey circle depicts the GC content on each strand.

Hangul mitogenome has two rRNA genes: 12s rRNA (located between tRNA^Phe^ and tRNA^Val^) and 16s rRNA (between tRNA^Val^ and tRNA^Leu^) (Fig. 1). The total length of these genes is 2,526 bp with an average positive AT skew (0.218) and negative GC skew (−0.097) (Table 1). In addition, it also contains 22 tRNA genes (total length of 1,515 bp) with length varying from 60 bp (tRNA^Ser^) to 75 bp (tRNA^Leu^) (Table 1). Most of these tRNAs (*n* = 14) are located on H-strand and majority (*n* = 21) showed the typical secondary cloverleaf structures. Leucine and Serine have two tRNAs each and tRNA^Ser^ (GCT) lacks the D-paired arm. The Control region (CR) is 917 bp long and located between tRNA^Pro^ and tRNA^Phe^. The base composition of CR was 31.7% T, 23.6% C, 29.4% A and 15.3% G with average negative AT (−0.038) and GC (−0.213) skew (Table 1).

### Phylogenetic analysis and divergence time estimate

**Figure 2:**
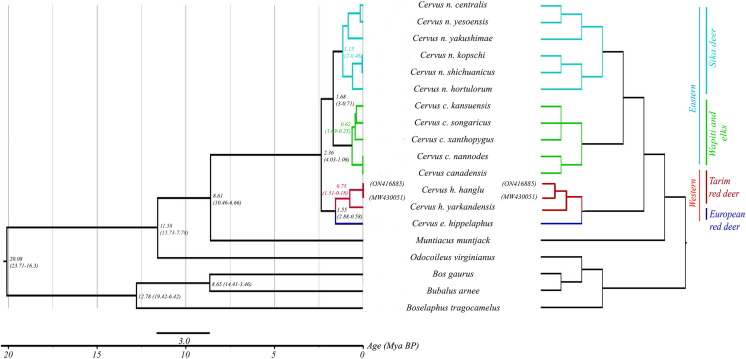
Phylogenetic relationship and assessment of divergence time in red deer species group. The right pane shows the phylogenetic relationship among different red deer species. For all nodes posterior probability values were ≥0.9. Three bovid species were used as outgroups. The left pane indicates the divergence times of this entire group. Node-specific ages are marked (with posterior probability values ≥0.9) along with the HPD intervals.

Mitogenome-based Bayesian phylogenetic reconstruction of the red deer complex (with 18 available species sequences including outgroups) reveals four mitochondrial lineages: *C. elaphus*, *C. hanglu*, *C. canadensis* and *C. nippon*. These lineages can be broadly divided into two major groups: the elaphoid clade (*C. hanglu* and *C. elaphus*) and the wapitoid clade (*C. canadensis* and *C. nippon*). The *C. elaphus* and *C. hanglu* forms the basal clade, followed by the *C. canadensis* and *C. nippon* as sister groups. The Hangul (*C. hanglu hanglu*) sequences (both generated in this study and the already available one) formed a distinct clade with its other subspecies *C. hanglu yarkandensis* and sister to the Hungarian red deer (*C. elaphus hippelaphus*). The tMRCA analysis suggested a divergence period spanning from 2.36 (Height Posterior Density (HPD) 4.23–1.06 million years ago (Mya)) to 0.16 (HPD 0.35–0.03 Mya) Mya (Fig. 2) for the red deer species complex. Our results indicated the divergence of the elaphoid group from the wapitoid group ~2.36 Mya (HPD 4.23–1.06 Mya), corresponding to the early Pleistocene period (Kuwayama & Ozawa, 2000). The Tarim deer species group separated ~1.55 Mya (HPD 2.88–0.58 Mya), overlapping with the Grünz glacial period in Europe (Kurten, 1968; Kuwayama & Ozawa, 2000). At Hangul group level, results suggest a coalescence of 0.75 Mya (HPD 1.51–0.18 Mya) between *C. hanglu* and *C. yarkandensis*.

The Hangul sequences generated from the male and female individuals were almost identical with five variable sites (*n* = 3 in PCGs and *n* = 2 in noncoding region). However, the Hangul mitogenome from GenBank showed 15 variable sites (*n* = 6 in the coding and *n* = 9 in the non-coding regions with three insertions in the rRNA region) with the sequences generated in this study. The mitogenomes of Hangul and closely related *Cervus h. yarkandensis* showed 345 variable sites. The highest difference was found with *Cervus n. yakushimae* (795 variable sites), but during annotation it was found that part of the mitogenome was missing and therefore was removed from further genetic distance analyses. While the protein coding regions among all the deer remained consistent (11,354 bp), major differences were found in the non-coding regions which varied from 913 bp for *Cervus h. yarkandensis* to 1,231 bp for *Cervus n. centralis*. One to seven base pair differences were seen for both tRNA (1,515–1,517 bp) and rRNA (2,525–2,532 bp) regions across the species (Table 1). As expected, the Hangul mitogenomes were genetically much closer (genetic distance = 0.001) than all other red deer species (genetic distance value varied from 0.022 to 0.052) (Table S1).

## Discussion

The Ministry of Environment, Forests and Climate Change, Government of India has listed Hangul as one of the 22 important Indian species that require immediate attention for future survival and recovery (https://wiienvis.nic.in/Database/SRP_8555.aspx). As part of this long-term initiative, the government has initiated a species recovery plan for this critically endangered species, especially focusing on creating a second population outside Dachigam national park (largest viable population), Jammu and Kashmir and establishing a conservation breeding facility. However, the conservation breeding program is facing challenges in selecting appropriate founder animals from the existing small and isolated hangul population in Kashmir (Ahmad & Nigam, 2014; Mukesh et al., 2015), specifically with limited knowledge on certain aspects of its biology including the patterns of genetic variation and evolutionary history. Earlier Hangul phylogeny research indicated contradictory patterns of its relationship with other species of the red deer group, mostly due to lack of data from Hangul. Comparative analyses of all published research on the species phylogeny suggest different outcomes. For example, Cytochrome b, control regions and whole mitogenome based phylogenetic research have shown similar sister-group relationships between the European red deer and Hangul (Ludt et al., 2004; Lorenzini & Garofalo, 2015; Meiri et al., 2018; Doan et al., 2018; Olivieri et al., 2014; Mackiewicz et al., 2022). However, Doan et al. (2022, complete cytochrome b) and Kumar et al. (2017, partial cytochrome b) (Kumar et al., 2017) have inferred Hangul as a basal group to the entire red deer complex. The most informative phylogenetic relationship among the Tarim red deers were presented by Lorenzini & Garofalo (2015) (cytochrome b and partial control region) and Meiri et al. (2018) (partial cytochrome b and control region) where all the species (*C. h. hanglu*, *C. h. bactrianus* and *C. h. Yarkandensis*) showed sister-group relationship with European red deer (Elaphoid group). Other studies involving *C. h. Yarkandensis* (closely related to *C. h. hanglu* and *C. h. bactrianus*) also showed similar relationship with the Elaphoid group (Doan et al., 2018; Mackiewicz et al., 2022). Based on genomic SNP markers, Hu et al. (2019) suggested a different phylogenetic relationship where Tarim deer (represented by *C. h. Yarkandensis*) formed a sister clade with *Cervus canadensis*, which was later explained as a result of frequent hybridisation amongst the Cervus group members (Heckeberg, 2020). Our complete mitogenome-based phylogeny supports the inferences arrived from the cytochrome b-based phylogeny as well as other studies involving four species of red deer (*C. nippon*, *C. elaphus*, *C. canadensis* and *C. hanglu*) (Zhang & Zhang, 2012; Frank et al., 2016; Li, Ba & Yang, 2016; Świsłocka et al., 2020; Mackiewicz et al., 2022). Given the support from the other studies, we believe that the mitogenome-based phylogeny of Hangul provides a clear relationship status within the Tarim red deer group. One noteworthy point is the non-availability of whole mitogenome sequence of *C. h. bactrianus* in this study, and when the mitogenome information is available it will provide the complete picture for the group’s phylogenetic relationship.

Another important outcome of this study is the assessment of Hangul divergence time within the existing Tarim red deer members (except *C. h. bactrianus*). The divergence period analyses are dependent on various factors such as molecular marker, mutation rate, node calibration points *etc*., Ritchie, Lo & Ho (2017). Overall, the red deer group divergence period varied between 7-1 Mya across different studies (Doan et al., 2022), probably due to differences in marker selection and analytical settings. For example, mitogenome-based divergence time estimation between European red deer and Tarim red deer (*C. h. Yarkandensis*) was found to be ~1.65 Mya (Frank et al., 2016) and ~1.88 Mya (Mackiewicz et al., 2022). Doan et al. (2018) reported a divergence period of ~1.54 Mya between the western (consisting of *C. elaphus* and *C. hanglu*) and the eastern (*C. nippon* and *C. canadensis*) red deer groups, whereas Zhang & Zhang (2012) along with Mackiewicz et al. (2022) suggested that ~2 Mya divergence period between the same groups. Our analyses provide a similar divergence period of ~2.36 Mya (4.03–1.06 Mya) between western and eastern groups of red deer. Within the western clade the Tarim deers diverged ~1.55 Mya from the European red deers (similar to Zhang & Zhang, 2012; Świsłocka et al., 2020 and Mackiewicz et al., 2022). Hangul diverged from *C. h. Yarkandensis* ~0.75 (1.51–0.18) Mya (supported by Meiri et al. (2018) where the most recent common ancestor of the *C. hanglu* group was reported as 0.23 Mya). These molecular dates were also supported by known paleobiogeographic events reported by other studies, specifically refugia during glaciations in the Pleistocene era (2.58–0.0117 Mya) (Kuwayama & Ozawa, 2000). Our divergence time estimates corroborate with known timelines of diversification and movement of four red deer lineages indicating their central Asian ancestry (Mahmut et al., 2002; Lorenzini & Garofalo, 2015; Frank et al., 2016; Mackiewicz et al., 2022). Diverged from a common ancestor, the eastern group (*C. nippon* and *C. canadensis*) moved towards south-eastern Asia and subsequently America *via* Berrings land bridges since pre-LGM times (1.68–0.16 mya) (Meiri et al., 2018). The western group, on the other hand, moved towards Middle East and then to Europe (Mahmut et al., 2002; Lorenzini & Garofalo, 2015; Kumar et al., 2017; Mackiewicz et al., 2022). The Tarim red deer (*C. h. yarkandensis*) separated from the European red deers due to geographic barriers (Taklimakan desert, Pamir plateau and then glacier covered Tein Shan mountains (Mahmut et al., 2002) ~1.55 Mya and adapted to the local arid habitats (Ababaikeri et al., 2020)). We speculate that Hangul might have migrated down towards the Hindu Kush Mountain ranges (~0.75 Mya), followed by upward movement (using the riparian corridors like it’s close related species *C. h. bactrianus* (Pereladova, 2013)) to its historical distribution across valleys of Chenab River to high altitudes of Kashmir. The Greater Himalayan (Zanskar mountain range) and Hindu Kush range (physical barrier) and the glaciation events during mid-Pleistocene could have acted as barriers, making it an endemic species to India. Further fossil evidences and historical genetic data are required to confirm this hypothesis.

The evolutionary history (in terms of diversification) of this species group is also evident in the mitogenome composition as well as genetic distance values. Hangul mitogenome sequence showed significant compositional similarities with the other species (Table 1). For example, while the length of the mitogenome varied between 16,351 bp (*C. h. hanglu* and *C. h. yarkandensis*) to 16,663 bp (*C. n. centralis*), majority of the length variations among these species were found in the control region. The total protein coding regions were identical in length amongst the red deer species (11,354 bp). Similarly, consistent amongst all red deers, except for the control region whole mitogenome, PCGs, tRNA and rRNA showed positive AT skew values, indicating an overall AT rich mitogenome. As expected, the phylogenetically close *C. h. yarkandensis* was genetically much closer than other red deer species (Table S1). Although earlier reports have suggested a low genetic variation in this species (Mukesh et al., 2015; Kumar et al., 2017), the genetic variability found in the three Hangul mitogenomes indicate more variations present within the species. As the origin of the available GenBank data is unknown, future studies need to collect more samples from different geographic locations to ascertain levels of genetic variation by generating mitogenome data. It is important to point out that only after extensive population-level study combining mitogenome and other nuclear markers (STR, SNPs *etc*.) it will be possible to identify suitable founder animals with appropriate genetic variations for any conservation breeding and reintroduction programs.

## Conclusion

The results of this study focus on the phylogenetic position of Hangul within the Tarim deer group and gives an insight over its evolutionary history in comparison to its sister species. We believe that wild Hangul mitogenome would act as a baseline information to augment our knowledge on its phylogeography and other population parameters and help in conserving this endemic and critically endangered species currently confined to the greater Dachigam landscape of Kashmir, India.

## Supplemental Information

10.7717/peerj.15746/supp-1 Supplemental Information 1 Genetic distance of *Cervus hanglu hanglu* in comparison to other red deer species.

10.7717/peerj.15746/supp-2 Supplemental Information 2 Raw sequence data for the Hangul individuals (*n* = 2).

10.7717/peerj.15746/supp-3 Supplemental Information 3 Annotated mitogenome of Hangul.

## Additional Information and Declarations

### Field Study Permissions

The following information was supplied relating to field study approvals (*i.e*., approving body and any reference numbers): All required permissions for fieldwork and sampling were provided by the Chief Wildlife Warden, Department of Wildlife Protection, Jammu and Kashmir (permit nos. WLP/TECH/116-19, WLP/TECH/871-74/2013, and WLP/F-101/Res/2019-20/424-27).

### Data Availability

The following information was supplied regarding data availability: The sequences are available at GenBank: ON416884 and ON416885.
