# Supplementary material for: Evolutionary insights on critically endangered Kashmir red deer or hangul (Cervus hanglu hanglu) through a mitogenomic lens
Source: PeerJ. 2023 Oct 19;11:e15746. doi: 10.7717/peerj.15746 (PMC10590573; doi:10.7717/peerj.15746)
Supplement: Supplemental Information 1 [file peerj-11-15746-s001.docx]

**Supplementary Table 1**: Genetic distance of *Cervus hanglu hanglu* in comparison to other red deer species

| **Species** | | **Common names** | **Accession no.** | **Genetic distance** | **No. of variable sites** | **Nucleotide diversity** |  |
| --- | --- | --- | --- | --- | --- | --- | --- |
|  |  |  |  |  |  |  |  |
| *Cervus h. yarkandensis* | | Yarkand deer | GU457435 | 0.022 | 345 | 0.021 |  |
| *Cervus e. hippelaphus* | | Hungarian red deer | KT290948 | 0.031 | 515 | 0.031 |  |
| *Cervus canadensis* | | Elk | MT534583 | 0.047 | 729 | 0.044 |  |
| *Cervus c. songaricus* | | Tian Shan wapiti | KJ025072 | 0.047 | 727 | 0.044 |  |
| *Cervus c. kansuensis* | | Gansu wapiti | NC039923 | 0.047 | 720 | 0.044 |  |
| *Cervus c. nannodes* | | Tule elk | MT430939 | 0.047 | 729 | 0.044 |  |
| *Cervus nippon hortulum* | | Dybowski's sika deer | KR868807 | 0.049 | 757 | 0.046 |  |
| *Cervus n.sichuanicus* | | Sichuan sika deer | JN389443 | 0.049 | 752 | 0.046 |  |
| *Cervus n. kopshi* | | South china sika deer | HQ832482 | 0.049 | 756 | 0.046 |  |
| *Cervus n. yakushimae* | | Yakushima sika deer | AB218689 | 0.052 | 795 | 0.048 |  |
| *Cervus n.yeonsis* | | Yezo sika deer | AB210267 | 0.051 | 787 | 0.048 |  |
| *Cervus n. centralis* | | Sika deer | NC006993 | 0.052 | 794 | 0.048 |  |
|  |  |  |  |  |  |  |  |
